# Docetaxel-induced radiation recall dermatitis with atypical features: A case report

**DOI:** 10.1097/MD.0000000000012209

**Published:** 2018-09-07

**Authors:** Masakuni Sakaguchi, Toshiya Maebayashi, Takuya Aizawa, Naoya Ishibashi

**Affiliations:** Department of Radiology, Nihon University School of Medicine, Oyaguchi Kami-cho, Itabashi-ku, Tokyo, Japan.

**Keywords:** docetaxel, radiation recall dermatitis, radiotherapy

## Abstract

**Introduction:**

Radiation recall dermatitis (RRD) associated with actinomycin D was first described by in 1959, followed by the reporting of several RRD-inducing drugs. In 1994, a study demonstrated docetaxel-induced RRD for the first time; however, despite some case studies reporting RRD, a little has been reported on it since then. Here we present a rare case of atypical docetaxel-induced RRD.

**Case presentation:**

The patient in his 60s was administered radiotherapy for high-risk prostate cancer. He continued receiving hormonal therapy for 2 years because of being in a high-risk group and became nadir. Six months since the completion of hormonal therapy, his prostate-specific antigen (PSA) level increased again. Based on the radiological examination, he was diagnosed with multiple lung, bone, and lymph node metastases. Accordingly, we started docetaxel (75 mg/m^2^) every 5 weeks in consideration of myelosuppression for hormone-resistant multiple metastases. Although lung metastasis shrunk by one cycle docetaxel, radiotherapy for the thoracic and lumbar vertebrae was performed for back pain and lumbago. On day 21, at the end of radiotherapy, the same dose of docetaxel was administrated for the third time. On day 7, after third docetaxel administration, erythema appeared in a irradiated field of the thoracic and lumbar vertebra. Erythema primarily appeared on the anterior side of the body, and no skin reaction was noted on the posterior part of the thoracic irradiated area. Notably, no skin reaction was observed in the previously irradiated field for prostate cancer.

**Conclusions:**

This case report draws attention to the development of atypical RRD after administration of docetaxel and advises careful follow-up even if RRD does not appear after the first docetaxel administration.

## 1 Introduction

Radiation recall phenomenon (RRP) is an acute inflammatory or tissue reaction that appears in a previously irradiated area and is induced by the administration of certain triggering drugs, such as chemotherapeutic agents. Radiation recall dermatitis (RRD) associated with actinomycin D was first described by D’Angio et al^[1]^ in 1959, followed by the reporting of several RRD-inducing drugs.^[2–4]^ In 1994, a study demonstrated docetaxel-induced RRD for the first time ^[5]^; however, despite some case studies reporting RRD, a little has been reported on it since then.^[6]^ Furthermore, the lack of comprehensive investigation of docetaxel-induced RRD renders its frequency and characteristics unclear. Docetaxel is widely used as a treatment regimen for head and neck, nonsmall cell lung, and prostate cancer.^[7]^ With a recent increase in docetaxel administration to treat several other types of cancer, both the frequency and incidence rate of RRD are anticipated to increase in the future. Here we present a rare case of atypical docetaxel-induced RRD and compare it with the findings of previous studies.

## 2 Case presentation

**Figure 1:**
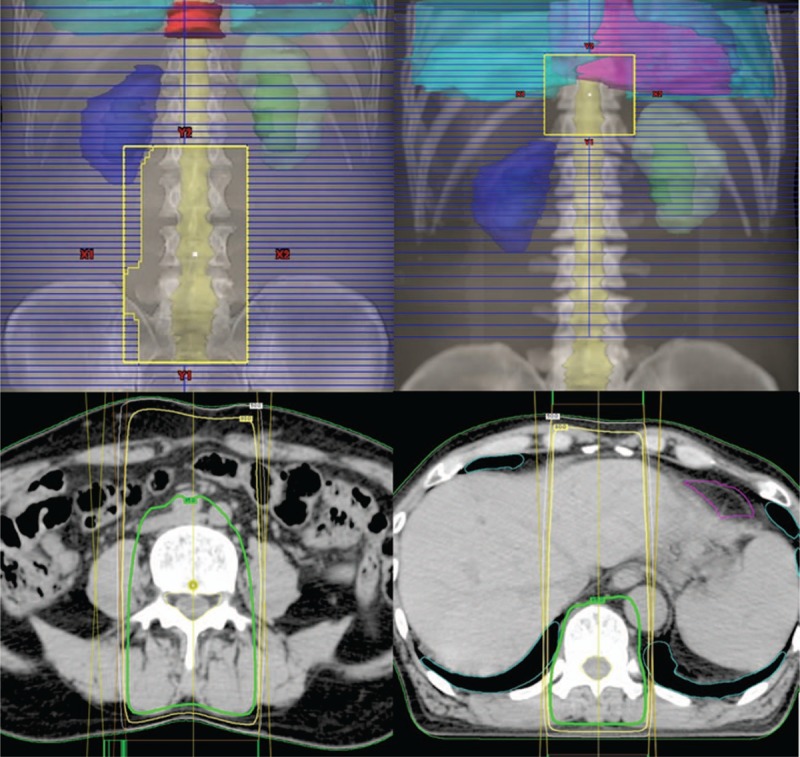
Beam's eye view of the anterior–posterior beam and dose distribution applied to the thoracic and lumbar vertebra. Bold, intermediate, and wire line represent 95%, 80%, and 50% dose, respectively.

**Figure 2:**
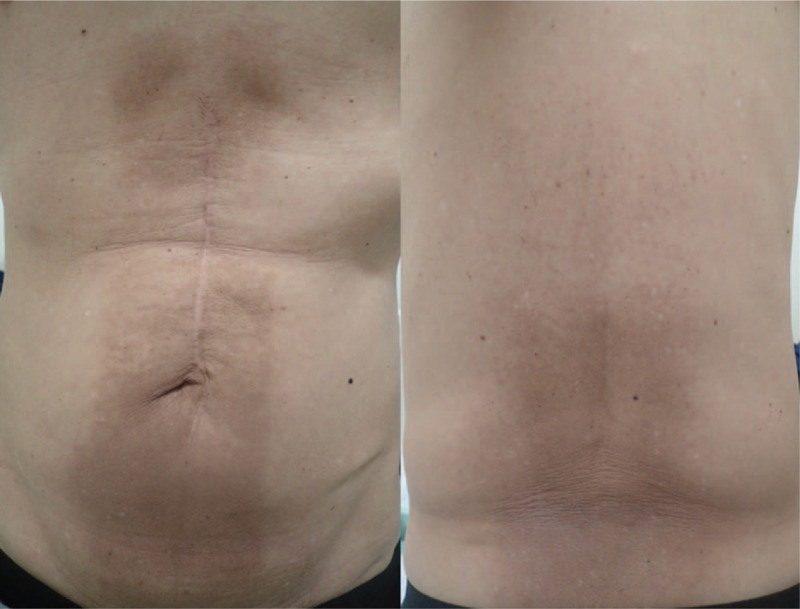
On day 7 after docetaxel administration, erythema appeared in the irradiation field. Erythema is more severe in the anterior skin than in the posterior skin; no erythema developed on the posterior skin of the thoracic part. Erythema border of the anterior skin is noticeable and entirely corresponding with the irradiated area.

We present the case of a man in his 60s who experienced atypical docetaxel-induced RRD. Although his medical history comprised a surgical intervention for a duodenum ulcer, he had no significant history of collagen vascular diseases. He was administered radiotherapy for high-risk prostate cancer after 1-year hormonal therapy (T1cN0M0 stage I; TNM 7th edition). Using 10-MV photons, he received 74 Gy in 37 fractions, 7 portals, and all coplanar irradiation. Although he experienced cystitis grade 1 (Common Terminology Criteria for Adverse Events 4.0.) as an adverse event, we observed no other adverse event. He continued receiving hormonal therapy for 2 years because of being in a high-risk group and became prostate-specific antigen (PSA) nadir. Six months since the completion of hormonal therapy, his PSA level increased again. Despite receiving hormonal therapy again, his PSA level continued increasing gradually. After 10 months of restarting hormonal therapy, he presented with dry cough and dyspnea. Based on the radiological examination, he was diagnosed with multiple lung, bone, and lymph node metastases. To distinguish primary lung cancer, biopsy was made from lung disease by bronchoscopy and established metastasis from prostate cancer. Accordingly, we started docetaxel (75 mg/m^2^) every 5 weeks for hormone-resistant multiple metastases. After one cycle of docetaxel, radiotherapy for the thoracic and lumbar vertebrae was performed for back pain and lumbago. In addition, irradiation was performed using 10-MV photon beams (anterior) and 10-MV (posterior) photon beams, 30 Gy in 10 fractions, and the anterior:posterior dose weight was approximately 1:2 (Fig. 1). Owing to a large irradiation field, we divided the radiation field into 2 parts: thoracic and lumbar vertebrae. Meanwhile, the same dose of docetaxel was concurrently restarted at the time of 18 Gy. We observed no adverse event during radiotherapy, and soon relief was achieved for back pain and lumbago. In addition, the PSA level decreased after the administration of docetaxel. On day 21, at the end of radiotherapy, the same dose of docetaxel was administrated for the third time. On day 7, after third docetaxel administration (day 28 from the end of radiotherapy), erythema appeared in an irradiated field of the thoracic and lumbar vertebra (Fig. 2). Erythema primarily appeared on the anterior side of the body, and no skin reaction was noted on the posterior part of the thoracic irradiated area. Notably, no skin reaction was observed in the previously irradiated field for prostate cancer. In fact, conservative treatment for RRD was performed using a skin care cream demonstrated better results by improving dermatitis. Subsequently, the patient underwent fourth docetaxel treatment without corticosteroid on day 28 after the third docetaxel administration; however, a less vivid skin reaction was noted this time compared with that noted during the previous session.

This retrospective study evaluated patient characteristics, diagnosis, and treatment retrieved from electronic medical records. All procedures followed the ethical guidelines of the 1964 Helsinki Declaration and its later amendments or comparable ethical standards. Written patient's consent to participate and ethical approval were waived for the retrospective study. Patients were informed before the start of treatment that data might be used in future investigations even if the outcome was death and all consented to the use of data.

## 3 Discussion

The frequency of RRD is approximately 5%–10%.^[8,9]^ Docetaxel-induced RRD is considered to be a comparatively rare condition than other drug-induced RRD. The most comprehensive research conducted by Mizumoto et al^[7]^ reported a frequency of docetaxel-induced RRD of 1.8% as the degree of rarity of this specific recall phenomenon.

Mizumoto et al^[7]^ also reported that radiation reaction on the skin and mucosal surface during radiotherapy might induce RRD because in their study, dermatitis and/or mucositis of >grade 2 developed during radiotherapy.^[7]^ Conversely, several studies have reported that acute skin reactions during radiotherapy are not always associated with subsequent RRD.^[10,11]^ Likewise, in our case, no dermatitis and no enterocolitis were reported during radiotherapy and until RRD developed. Camidge and Price^[12]^ reviewed previous reports on the RRD mechanism and listed vascular damage, epithelial stem cell inadequacy, and epithelial stem cell sensitivity as the mechanism. Although the underlying mechanism of RRD remains unclear, it might be attributed to different mechanisms ranging from radiation dermatitis to radiotherapy. Our case draws attention to the development of RRD and suggests a careful follow-up even if no skin reaction is detected during radiotherapy.

Mostly previous studies have reported that RRD occurs with lower energy photon beams,^[13,14]^ and RRD with 10-MV photon beam, as observed in our patient, is relatively rare. Radiation doses at the skin surface with the 10-MV photon beam were less than 6-MV photon beams. Yeo et al^[14]^ calculated a different part of 4 threshold doses and estimated that a radiation dose between 16.8 and 18.7 Gy induced RRD in their case. Stelzer et al^[4]^ reported patients with cutaneous Kaposi sarcoma who developed RRD by bleomycin. Although the recall-triggering drug was administrated at different doses (40, 20, or 8 Gy), only the area treated with 40 Gy developed RRD. Regarding these studies, the administered radiation dose seems to play a crucial role in the development of RRD. Perhaps, a comparatively lower dose might not induce RRD or cause a mild reaction. However, our case reported an atypical appearance at this hypothesized radiation dose. Our radiotherapy consisted of 2 anterior–posterior portals, and the posterior portal had approximately 2 times the dose weight compared with that of the anterior portal; the radiation dose of the skin was higher in the posterior skin than in the anterior skin. However, RRD was more severe on the anterior skin than on the posterior skin, in particular, the posterior skin of the thoracic part did not develop RRD. Sears ^[15]^ reported hydroxyurea-induced RRD in 2 treated fields, but it was observed 8 days earlier in a field that had received a higher dose. In our case, we continued the follow-up for a while, but did not detect RRD on the posterior thoracic part. In our experience, RRD might appear despite low skin dose, unlike that reported previously. No RRD developed irrespective of a high radiation dose (posterior thoracic part) in our patient, raising skepticism on the correlation between a dose of radioactivity and its appearance.

RRP, including dermatitis and stomatitis, indicates that this phenomenon tends to develop approximately 1 week after the administration of triggering agents,^[1,12,16]^ whereas RRP occurred within 7 days in our patient, as reported previously. The longest interval between radiotherapy and RRD is 7 years.^[17]^ Our patient received radiotherapy for primary prostate cancer 2 years ago, when RRD could also have developed in the lower pelvic site of the skin but did not develop. Radiation dose is similar to that during radiotherapy for bone metastases, RRD developed at this radiotherapy for bone metastases but did not develop during the previous radiotherapy for prostate cancer. Although the mechanism is unknown, we hypothesize that the skin surface that receives radiation once, recovers with time, and it becomes difficult of RRD to occur at the same dose.

Usually, RRD occurs after the first exposure to a recall-triggering drug. In contrast, Castellano et al^[18]^ reported a case in which gemcitabine was administered without a effect on the skin on day 1, but RRD was reported after the day 8 dose. In our case, the first administration of docetaxel at 18-Gy radiation schedule reported no adverse skin reaction. These results support the presence of RRD.

In our case, successful re-administration of docetaxel without recurrence of RRD occurred without reducing the docetaxel dose and not using a steroid. Regarding the re-administration of docetaxel after the appearance of RRD once, a dose reduction of the trigger drugs, using oral steroids, or re-challenging the administration on the same schedule have been reported, but their efficacy remains unproven,^[19]^ thereby necessitating further investigation.

## 4 Conclusion

Although several hypotheses on drug hypersensitivity reactions, with or without additional radiation-induced cellular sensitivity to such reactions, have been described, its underlying mechanism remains unclear. In our case, the atypical feature was different from that reported previously. This case report draws attention to the development of atypical RRD that warrants further investigation in the future.

## Author contributions

**Conceptualization:** Takuya Aizawa, Naoya Ishibashi, Masakuni Sakaguchi.

**Data curation:** Masakuni Sakaguchi.

**Methodology:** Masakuni Sakaguchi.

**Supervision:** Toshiya Maebayashi.

**Writing – original draft:** Masakuni Sakaguchi.

**Writing – review & editing:** Masakuni Sakaguchi, Toshiya Maebayashi, Takuya Aizawa, Naoya Ishibashi.

Masakuni Sakaguchi: 0000-0002-7204-0701.
